# Study on Preparation and Grinding Performance of Vitrified Bond CBN Grinding Wheel with Controllable Porosity

**DOI:** 10.3390/mi14010021

**Published:** 2022-12-22

**Authors:** Qiao Xu, Honggen Zhou, Hengheng Wu, Li Sun, Xiaona Shi, Guochao Li

**Affiliations:** School of Mechanical Engineering, Jiangsu University of Science and Technology, Zhenjiang 212100, China

**Keywords:** vitrified bond CBN grinding wheel specimens, controllable porosity, grinding force, grinding temperature, surface roughness

## Abstract

Vitrified bond cubic boron nitride (CBN) grinding wheel specimens with controllable porosity were prepared by regulating the pore former dextrin content and varying the forming pressure, and the performance of the grinding camshaft was studied. The porosity of the specimens increases with the increase in dextrin content, and decreases first and then increases with the increase in the forming pressure. The grinding experiments show that the dextrin content is negatively correlated with the grinding force and grinding temperature, while the grinding force and grinding temperature of the specimens increase and then decrease with the increase in the forming pressure. When we observe and measure the grinding surface of the specimen and workpiece, we see that the surface roughness of the specimen after grinding is smaller than that before grinding. In addition, the greater the porosity of the specimen, the rougher the surface of the workpiece after grinding.

## 1. Introduction

The camshaft, as one of the key engine components, serves to control the opening and closing of the intake and exhaust valves [1,2]. Camshafts usually operate at high speeds and under complex force conditions, and need to withstand certain torques and periodic shock loads [3]. Therefore, high demands are placed on the machining process of camshafts.

The vitrified bond cubic boron nitride (CBN) grinding wheel is one of the most promising abrasive tools because of its low grinding force, good heat resistance, self-sharpening, and long life [4,5,6]. It has been widely used in the grinding of difficult workpieces such as steel, cast iron and alloy, ranging from high-efficiency grinding to high-precision grinding [7,8,9]. Compared with the metal and resin grinding CBN wheel, the vitrified bond CBN wheel exhibits higher bond strength and better self-dressing capabilities [5,10]. In addition, the vitrified bond grinding wheel can be adjusted in a wide range of porosities by changing the formulation and manufacturing process [11]. Pores play a key role in the grinding process, especially the interconnected pores [12]. First, they can provide channels for a coolant flow to the work area and reduce the heat generated during grinding, which contributes to the quality of workpiece machining as well as the life of the grinding wheel [13]. Secondly, they can additionally provide space for debris to be removed from the work area and prevent clogging of the grinding wheel [14]. In addition, pores have a direct impact on the strength, hardness, and grinding efficiency of the vitrified bond grinding wheel, which can be effectively adjusted to meet the demands of different machining conditions [15,16]. Therefore, in this paper, a vitrified bond CBN grinding wheel was used to grind the camshaft.

Pores in a vitrified bond wheel can be controlled either by adjusting the forming pressure or by adding the pore former [17]. In the absence of a pore former, the porosity in the vitrified bond wheel is changed by changing the size of the forming volume. In this way, a larger porosity cannot be obtained, although it is not easy to obtain a large pore size. If the pore former is used, the number, morphology and distribution of pores in the vitrified bond wheel are controlled by varying the amount and size of the added pore former and the way it is added [18]. Different pore formers have been reported by different researchers, such as polymethyl methacrylate (PMMA) [19], aluminum powder [20], dextrin [21], granulated sugar [22] and graphite [23]. In this paper, dextrin was chosen as the pore former due to its low ash content and its ability to reduce the contamination of ceramic materials by alkali and trace element ions. Firstly, the effect of the dextrin content and forming pressure on the porosity of vitrified bond CBN grinding wheels was investigated. Subsequently, experiments were carried out to obtain the effect of the dextrin addition and forming pressure on the grinding force and grinding temperature when grinding camshafts, and then verified and analyzed by observing the surface morphology of the vitrified bond CBN grinding wheels after grinding. In addition, the surface morphology of the vitrified bond CBN grinding wheels before and after grinding was analyzed, as well as a comparison of their surface roughness. Similarly, the surface morphology of the grinding camshaft was analyzed, and the surface roughness was compared.

## 2. Experimental Details

For the convenience of research, the relevant experimental analysis was performed by making a vitrified bond CBN grinding wheel specimen instead of a grinding wheel in this paper.

### 2.1. Preparation of Vitrified Bond

A R_2_O–RO–B_2_O_3_–Al_2_O_3_–SiO_2_ glass system was selected to prepare the vitrified bond. The composition of the vitrified bond is shown in Table 1. The raw materials were mixed and fritted in an alumina crucible at 1400 °C for 60 min, then the frit was put into water for quenching. The frit was subsequently crushed, and ball milled with a ball mill. After the ball milling was completed, the glass powder was taken out and passed through a 120-mesh sieve.

### 2.2. Preparation of Vitrified Bond CBN Grinding Wheel Specimens

**Figure 1:**
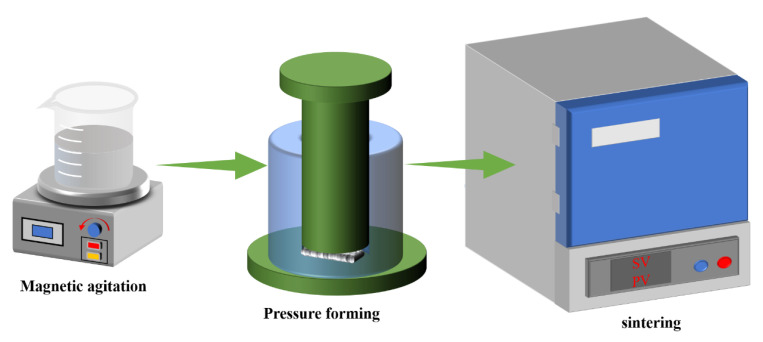
Manufacturing process of vitrified bond CBN grinding wheel specimen.

**Figure 2:**
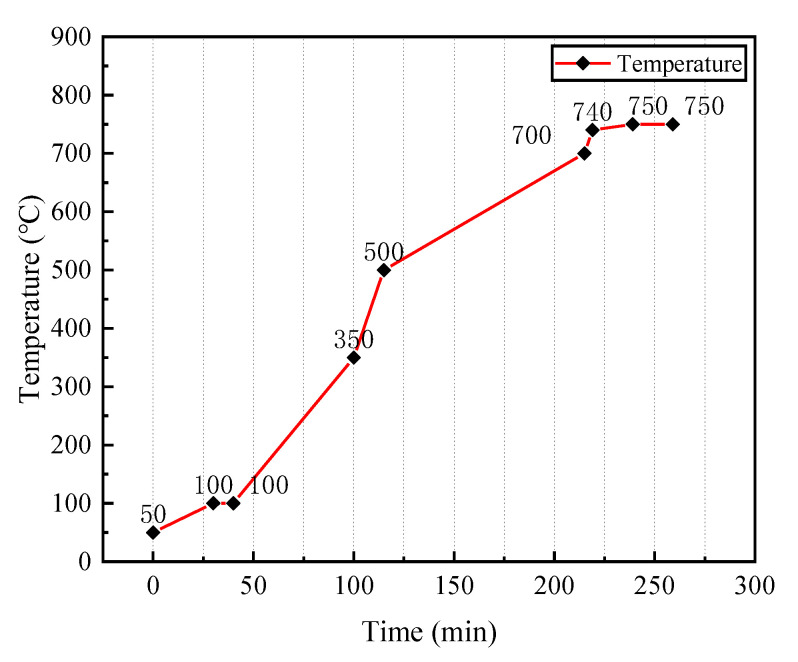
Sintering temperature curve.

**Figure 3:**
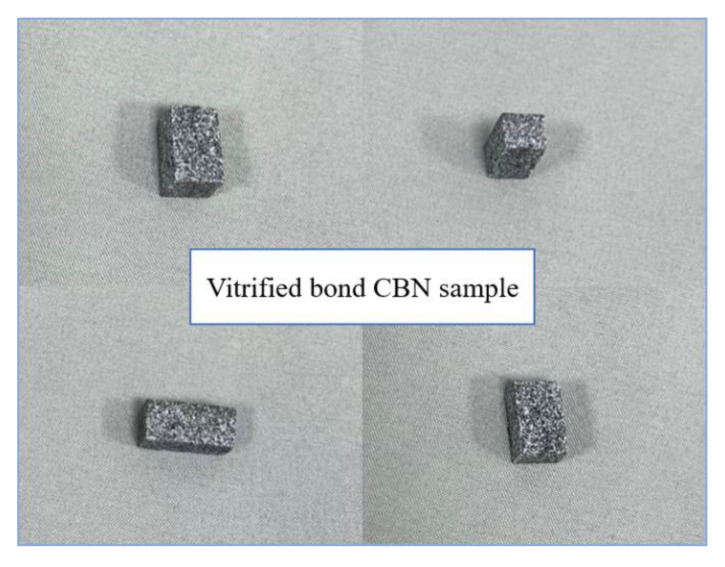
Appearance of vitrified bond CBN grinding wheel specimen.

The preparation process of the vitrified bond CBN grinding wheel specimens is shown in Figure 1. The operation is as follows: 120/140 mesh CBN abrasive grains (single crystal, Zhongnan Jiete Superabrasives Co., Ltd., Zhengzhou, China) were taken and cleaned in hydrochloric acid to remove impurities on the surface. The vitrified bond, CBN abrasive grains and dextrin were divided into four groups according to the ratio in Table 2. The materials in each group were mixed well with an automatic machine and then pressed into the mold (20 mm × 10 mm × 10 mm), and three blanks were made in each group. The blanks were then sintered in a tube atmosphere furnace, and the sintering curve is shown in Figure 2. As can be seen from the figure, these blanks were first heated to 100 °C and held for 10min. They were subsequently heated to 750 °C and kept for 10 min. Finally, they were cooled to room temperature. The constant temperature processes are set at 100 °C and 750 °C, respectively. The purpose of the constant temperature at 100 °C is to remove the moisture from the vitrified bond, which prevents a large porosity due to the violent vaporization of moisture during the subsequent temperature increase. The purpose of the constant temperature at 750 °C is to liquefy the vitrified bond sufficiently so that it can better encapsulate the CBN abrasive particles. After the sintering was completed, the specimens were taken out and the appearance of the morphology is shown in Figure 3.

According to experimental requirements, the amount of dextrin added was fixed and the production of specimens under different forming pressures was carried out. The blanks were pressed and formed according to the forming pressures shown in Table 3. Three blanks were made for each of the different forming pressures and the sintering process was the same as above.

### 2.3. Test Experiment

The porosity was measured as follows: using distilled water as the medium, we measured the porosity of the specimens by the Archimedes drainage method and took the average value of the porosity of three identical specimens.

**Figure 4:**
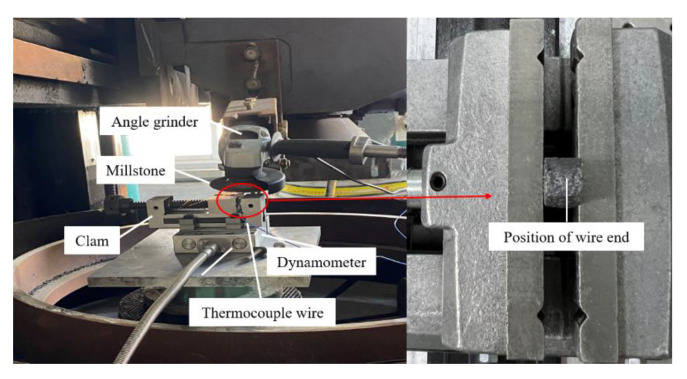
Grinding force and grinding temperature measuring device.

The grinding experiment was carried out as follows: as shown in Figure 4, an angle grinder with a speed of 11,000 r/min was fixed on the grinding machine, and a camshaft grinding disc of 100 mm diameter made of 40 Cr alloy steel was then installed on the angle grinder. The clamp was installed on the force sensor KISTLER 9129AA (Kistler Instrumente AG, Winterthur, Switzerland), and the specimen was fixed using the fixture. The measuring end of the armored thermocouple was fixed at the grinding position, and the other end of the thermocouple was connected to the UT320A thermometer. During the experiment, the grinding disc was rotated in contact with the specimen and the grinding temperature and grinding force were measured by the thermocouple and the force sensor. A large depth of field digital optical microscope OLYMPUS-1000 (Olympus, Shinjuku, Japan) was used to observe the surface morphology of the specimen before and after grinding and of the grinding workpiece, and their surface roughness was also measured.

## 3. Results and Discussion

### 3.1. Effects of Dextrin Addition and Forming Pressure on Porosity of Vitrified Bond CBN Grinding Wheel Specimens

**Figure 5:**
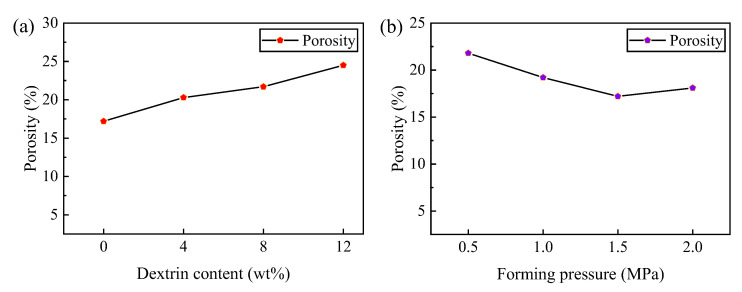
Effects of (**a**) dextrin content and (**b**) forming pressure on the porosity of the vitrified bond CBN grinding wheel specimens.

As shown in Figure 5a, the porosity of the vitrified bond CBN grinding wheel specimens increases from 17.2% to 24.5% with the increasing dextrin content. The reason for this situation is mainly due to the gradual carbonization of dextrin under high temperature conditions and the reaction with oxygen to produce gas. This causes the dextrin itself to shrink continuously, and the expansion and escape of the gas eventually contribute to the formation of pores. Therefore, the dextrin content increases as does the porosity.

To obtain the relationship between the forming pressure and porosity of the specimens, the dextrin addition was fixed at 8 wt% and analysis of the porosity variation pattern was carried out. As shown in Figure 5b, the porosity of the vitrified bond CBN grinding wheel specimens decreases from 21.8% to 17.2% in the range of the forming pressure from 0.5 MPa to 1.5 MPa. However, when the forming pressure is increased to 2 MPa, the porosity of the specimens increases from 17.2% to 18.1%. The reason for this phenomenon is that as the forming pressure increases initially, the specimen becomes denser, reducing the size of the pores in the specimen while the porosity of the specimen decreases. When the vitrified bond CBN specimen blank is too tight, the gas generated in the sintering is blocked in the specimen and cannot initially escape. As the gas content increases, the internal pressure exceeds the tolerance limit of the surrounding vitrified bond, resulting in increased porosity and even damage to the nearby vitrified bond such as cracks. The porosity of the specimen also increases.

### 3.2. Effect of Dextrin Content on Grinding Force and Grinding Temperature of Vitrified Bond CBN Grinding Wheel Specimens

**Figure 6:**
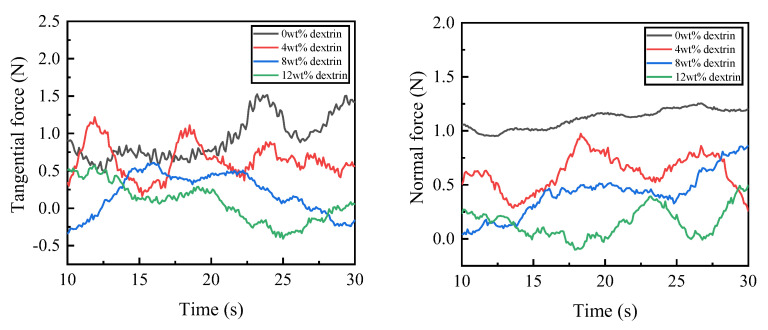
Effect of dextrin addition on the grinding force of the vitrified bond CBN grinding wheel specimens.

**Figure 7:**
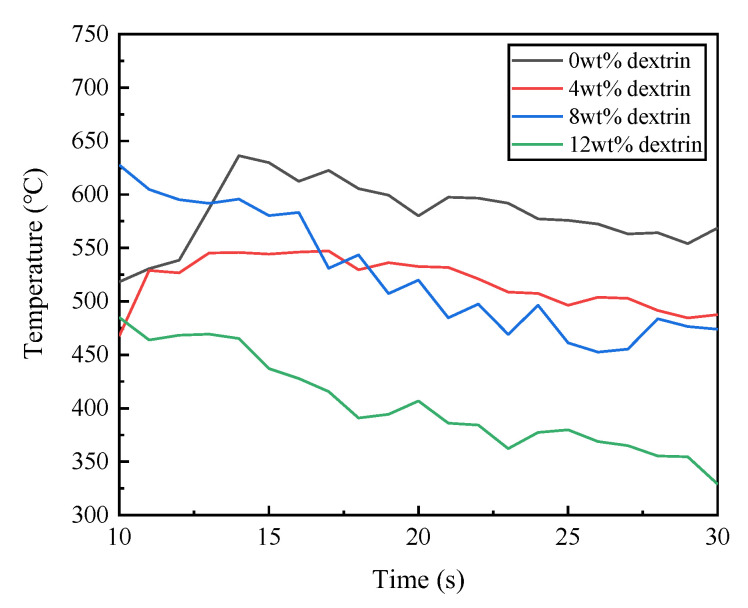
Effect of dextrin addition on the grinding temperature of vitrified bond CBN grinding wheel specimens.

During the experiment, there was an obvious vibration phenomenon at the beginning and end of the grinding. In order to ensure the stability of the measured values, the grinding force values in the 10–30 s interval were taken for graphing and smoothed. Because the tangential force and normal force can better reflect the change in grinding force in the grinding process, the tangential force and normal force are selected as the research objects. Figure 6 shows the graph of the influence of the dextrin addition on the grinding force. As can be seen from the figure, the overall grinding force of the specimen is larger without the addition of dextrin, and gradually decreases with the increase in the dextrin content. Figure 7 shows the graph of the grinding temperature variation between 10 s and 30 s during the experiment. It can be seen from the figure that the grinding temperature of the specimen is relatively high when no dextrin is added, while the grinding temperature shows a decreasing trend as the amount of dextrin addition increases. 

The reason for the above changes in the grinding force and temperature is that the addition of the dextrin increases the porosity of the specimen, which helps to contain and dissipate the chips and reduces the load and resistance during grinding. As a result, the grinding force and grinding temperature gradually decrease with the addition of dextrin. 

**Figure 8:**
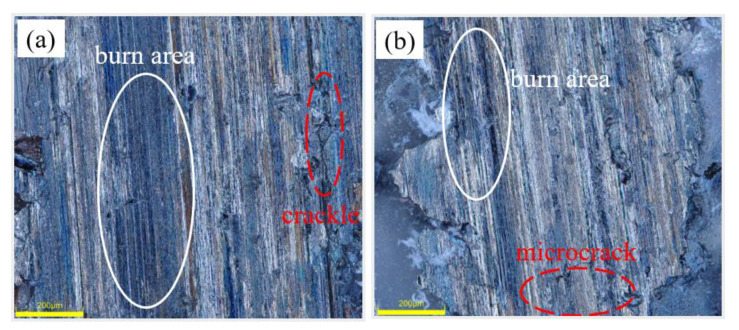
Morphology of grinding surfaces of vitrified bond CBN grinding wheel specimens with different dextrin additions (**a**) 0 wt% (**b**) 12 wt%.

Figure 8 shows the surface morphology of the specimens after grinding the camshaft grinding disc with 0 wt% and 12 wt% dextrin added. As can be seen from Figure 8a, the surface of the specimen without the addition of dextrin produces larger cracks and a wider range of narrow bands of black burns. In contrast, in Figure 8b, it can be seen that only minor cracks exist on the surface of the specimen with the addition of dextrin, and the black range of the burns is narrower. The difference mentioned above is due to the poor heat dissipation ability of the specimens when no dextrin is added. When the grinding wheel is grinding, the speed relative to the workpiece is high, generating intense external friction with the surface of the workpiece as well as heat. Because the cutting of each abrasive is instantaneous, the heat generation is also instantaneous and cannot be dissipated in time; therefore, the instantaneous temperature in the grinding area is high. If the heat dissipation capacity is not good, it will easily cause burns on the grinding surface and the burned area will appear black. The more severe the burn, the greater will be the ability to damage the tissue and the greater the cracks produced will be. This also indicates that the grinding temperature of the specimen with the addition of dextrin is lower than when we were grinding camshaft grinding discs without the addition of dextrin. 

### 3.3. Effect of Forming Pressure on Grinding Force and Grinding Temperature of Vitrified Bond CBN Grinding Wheel Specimens

Because the grinding force and grinding temperature are lowest when 12 wt% dextrin is added, a vitrified bond CBN grinding wheel specimen with 12 wt% dextrin was chosen in the selection for the related research into the forming pressure.

**Figure 9:**
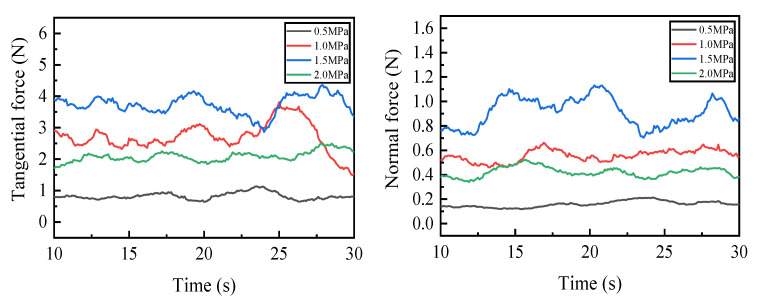
Grinding force of vitrified bond CBN grinding wheel specimens under different forming pressures.

**Figure 10:**
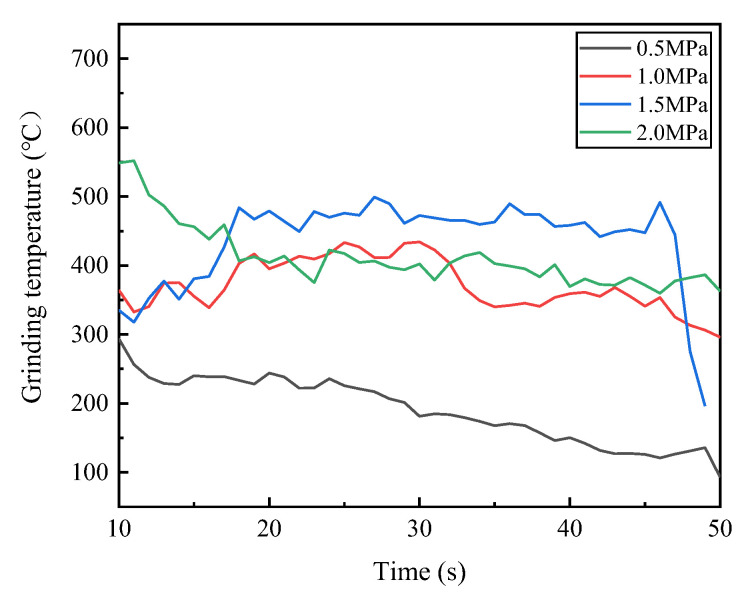
Grinding temperature of vitrified bond CBN grinding wheel specimens under different forming pressures.

Figure 9 shows the trend of the grinding force when we were grinding camshaft grinding discs with vitrified bond CBN grinding wheel specimens prepared under different forming pressures. It can be seen that when the forming pressure is 0.5 MPa, the grinding force is at its minimum. As the forming pressure increases to 1.5 MPa, the grinding force generally tends to be increased despite the crossover phenomenon of the grinding force signal. However, when the forming pressure increases to 2 MPa, the grinding force decreases. Figure 10 shows the graph of the grinding temperature variation between 10 s and 30 s during the experiment. It can be seen from the figure that the grinding temperature is at its lowest when the forming pressure is 0.5 MPa. As the forming pressure increases to 1.5 MPa, the grinding temperature generally shows an increasing trend. However, when the forming pressure increases to 2 MPa, the grinding temperature decreases relatively.

The reason why the grinding force and grinding temperature are mentioned above is: the lower the forming pressure, the lower the density of the sintered specimen and the larger the porosity of the specimen, resulting in a lower grinding force and lower grinding temperature. Therefore, when the forming pressure is between 0.5 MPa and 1.5 MPa, the grinding test shows that the grinding force and grinding temperature increase with the increasing forming pressure. However, when the forming pressure is increased to 2 MPa, first of all, the elastic deformation between the vitrified bond and dextrin particles leads to a higher residual stress in the blank, which causes swelling and microcrack damage in the sample during sintering; secondly, the blank is so compact that the gas formed by the dextrin at a high temperature cannot easily escape, and remains inside and increases the volume of the pores due to gas expansion. On the other hand, the gas that fails to escape increases the internal stress on the specimen, making it more susceptible to developing microcracks. Therefore, too large a forming pressure increases the porosity of the specimen, which in turn reduces the grinding force and grinding temperature of the vitrified bond CBN grinding wheel specimen against the camshaft grinding discs.

**Figure 11:**
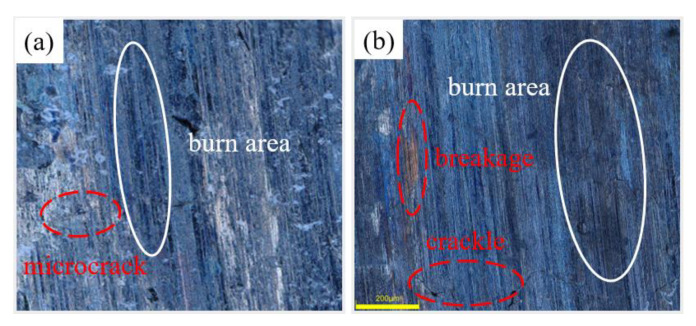
Morphology of grinding surfaces of vitrified bond CBN grinding wheel specimens under different forming pressures (**a**) 0.5 MPa (**b**) 1.5 MPa.

Figure 11 shows the surface morphology of the specimens prepared by grinding camshaft grinding discs at forming pressures of 0.5 MPa and 1.5 MPa. Under the forming pressure of 0.5 MPa, small cracks appear on the grinding surface, and there are small-scale lumpy burns. However, under the forming pressure of 1.5 MPa, large cracks appear on the grinding surface with breakage and extensive burns. It was additionally proved that the grinding force and grinding temperature of the vitrified bond CBN grinding wheel specimen decrease with the increase in the forming pressure in the range of 0.5 MPa to 1.5 MPa.

### 3.4. Surface Morphology of Vitrified Bond CBN Grinding Wheel Specimens and Camshaft Grinding Discs 

#### 3.4.1. Surface Morphology of Vitrified Bond CBN Grinding Wheel Specimens before and after Grinding

**Figure 12:**
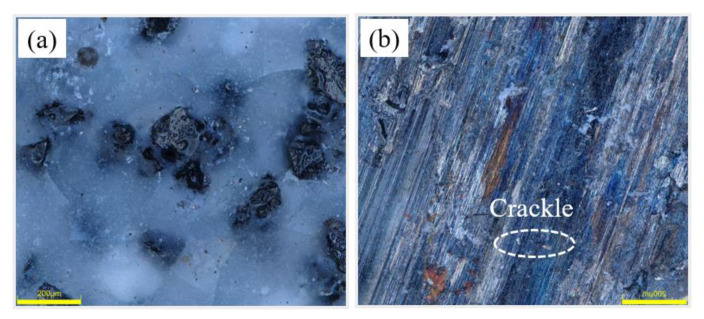
Surface morphology of the vitrified bond CBN grinding wheel specimen (**a**) before grinding (**b**) after grinding.

**Figure 13:**
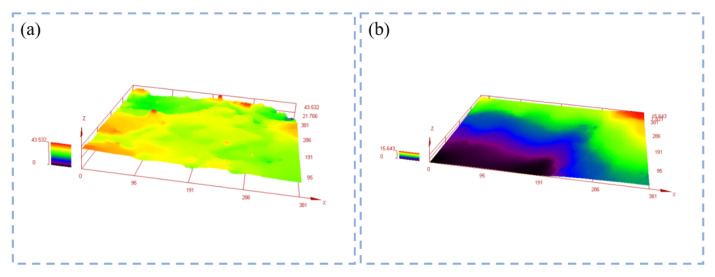
Three-dimensional morphology height of the vitrified bond CBN grinding wheel specimen (**a**) before grinding (**b**) after grinding.

**Figure 14:**
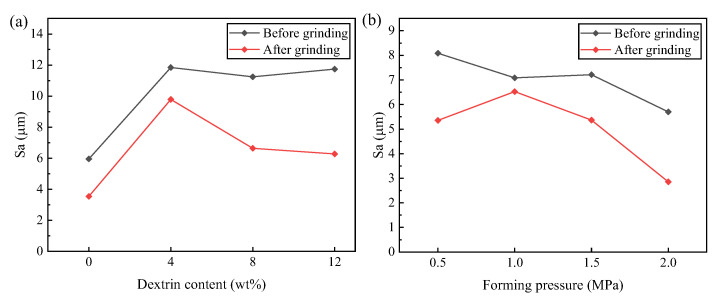
Surface roughness of vitrified bond CBN grinding wheel specimens before and after grinding (**a**) under different dextrin addition (**b**) under different forming pressure.

Figure 12 is a morphology comparison diagram of the specimen before and after grinding. Before grinding, the CBN abrasive grains maintain their original shape, the vitrified bond holds the CBN abrasive grains, and the abrasive grains are evenly distributed with the pores. After grinding, some pores on the surface of the specimen are covered by debris, and some CBN abrasive grains are flattened to reveal almost the same height, with the result that the surface of the specimen is flatter. Figure 13 shows the height of the three-dimensional morphology of the specimen before and after grinding. It can be seen that the original surface height of the specimen is higher, while the height between the surface after grinding is lower compared with that before grinding, and the surface is flatter. Figure 14 shows the surface roughness of the specimens before and after grinding. Figure 14a shows the surface roughness values of the specimens before and after grinding with different dextrin contents, while Figure 14b shows the surface roughness values of the specimens before and after grinding with different forming pressures. It can be seen that the surface roughness of the specimens after grinding is smaller than that before grinding, regardless of the grinding of the specimens with different dextrin content or the grinding of the specimens with different forming pressures. At the same time, the additional amount of dextrin and the forming pressure have little influence on the surface roughness of the specimens before and after grinding, and the values of the surface roughness are in the range of 2–12 μm.

#### 3.4.2. Surface Morphology of Camshaft Grinding Discs

**Figure 15:**
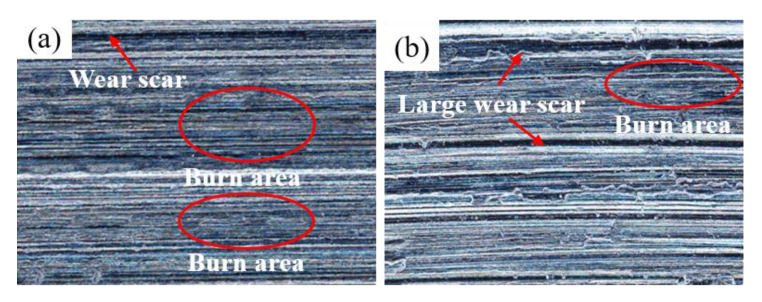
Surface morphology of camshaft grinding discs after grinding of vitrified bond CBN grinding wheel specimens with different dextrin additions (**a**) 0 wt% dextrin (**b**) 12 wt% dextrin.

**Figure 16:**
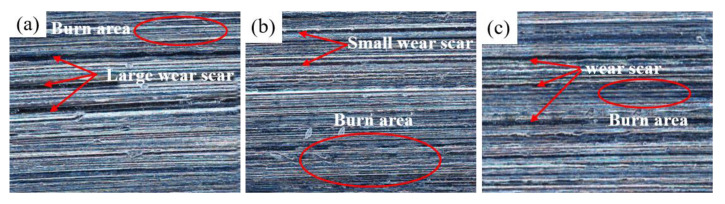
Surface morphology of camshaft grinding discs after grinding of vitrified bond CBN grinding wheel specimens with different forming pressures (**a**) 0.5 MPa (**b**) 1.5 MPa (**c**) 2 MPa.

Figure 15 shows the surface morphology of the camshaft grinding disc after the grinding of vitrified bond CBN grinding wheel specimens subject to different dextrin additions. It can be seen in the figure that with the addition of dextrin, the range of burns on the surface of the camshaft grinding disc becomes smaller, while the surface wear scars are deeper. When we measure the surface roughness of the grinding surface, we see that the surface roughness without the addition of dextrin is 0.056 μm, while the surface roughness of the grinding surface with the addition of 12 wt% dextrin is 0.12 μm. It shows a smoother surface without the addition of dextrin. Figure 16 shows the surface morphology of the camshaft grinding disc after the grinding of vitrified bond CBN grinding wheel specimens under different forming pressures. As can be seen in the figure, when the forming pressure is 0.5 MPa, the wear scars on the grinding surface are deeper, but the surface burns are less extensive. As the forming pressure increases to 1 MPa, the wear scar depth on the grinding surface becomes shallow, while the surface burn area expands. When the forming pressure is increased to 2 MPa, the wear scar depth deepens slightly and the surface burn range decreases. The surface roughness of the grinding surface at a forming pressure of 0.5 MPa is measured to be 0.156 μm. As the forming pressure increases to 1.5 MPa, the surface roughness changes to 0.042 μm, while at a forming pressure of 2 MPa, the surface roughness is 0.069 μm. As a result, the surface is smoother at a forming pressure of 1.5 MPa.

The reason for the above phenomenon is related to the porosity. When the porosity increases, it is easier for the abrasives to be exposed and to have more space to hold the chips, while it is less likely that the abrasives involved in grinding will be buried by the chips. As a result, the workpiece has deeper wear scars and a larger surface roughness. The reason for the reduced burn is that the increased porosity helps to dissipate heat. In summary, it can be concluded that the greater the porosity, the rougher the grinding surface.

## 4. Conclusions

In conclusion, we proposed a method for changing the porosity of a vitrified bond CBN grinding wheel by adding dextrin and adjusting the forming pressure. Firstly, the relationship between the dextrin content, forming pressure and porosity of the specimens was investigated. The porosity increased with the increase in dextrin content, and first decreased and then increased with the increase in forming pressure. Subsequently, the effects of the dextrin content and forming pressure on the grinding force and grinding temperature of the specimens for the grinding of camshaft grinding discs were investigated. With the increase in dextrin content, the grinding force and grinding temperature of the specimens gradually decreased. With the increase in forming pressure, the grinding force and grinding temperature of the specimens first increased and then decreased. When the forming pressure was 1.5 MPa, the grinding force and grinding temperature reached their maximum. Finally, the surface morphology of the specimens before and after grinding and the camshaft grinding discs after grinding were investigated. The surface roughness of the specimens after grinding was smaller than that before grinding. The greater the porosity, the rougher the camshaft grinding disc.

## Figures and Tables

**Table 1:** Composition of vitrified bond.

Composition	SiO_2_	Al_2_O_3_	B_2_O_3_	Na_2_O	Li_2_O	ZnO	MgO
*wt*%	55	15	10	7	3	5	5

**Table 2:** Vitrified bond CBN grinding wheel specimens with different dextrin contents.

Sample	Vitrified Bond/g	CBN Abrasive/g	Dextrin/g
1	2	0.6	0
2	2	0.6	0.8
3	2	0.6	0.16
4	2	0.6	0.24

**Table 3:** Vitrified bond CBN grinding wheel specimens with different forming pressures.

Sample	Vitrified Bond/g	CBN Abrasive/g	Forming Pressure/MPa
5	2	0.6	0.5
6	2	0.6	1
7	2	0.6	1.5
8	2	0.6	2

## Data Availability

The data presented in this study are available from the corresponding author upon reasonable request.
